# Batteries Included: A Case of Multiple Self-Inflicted Foreign Bodies in the Lower Genitourinary Tract

**DOI:** 10.7759/cureus.73048

**Published:** 2024-11-05

**Authors:** Omar Brijawi, Evan Hartman, Steven Wright, Noah R Schneegurt

**Affiliations:** 1 Internal Medicine, Mount Carmel Health System, Grove, USA; 2 Internal Medicine, Wright State University Boonshoft School of Medicine, Dayton, USA; 3 Internal Medicine, University of Wisconsin–Madison, Madison, USA

**Keywords:** ct, cystoscopy, foreign body insertion, lower urinary tract, urinary bladder

## Abstract

Self-insertion of foreign objects into the lower urinary tract is an uncommon presentation with diverse underlying motivations, including psychiatric illness, sexual gratification, intoxication, and perceived contraception. A wide variety of objects may be inserted, leading to varied symptom presentations. We report the case of a 51-year-old male with a history of post-traumatic stress disorder, antisocial personality disorder, and prior self-mutilation, who presented to the ED following self-insertion of multiple foreign objects into the lower urinary tract. CT revealed an AAA battery lodged in his urethra alongside paper clips, with toothpaste caps found within his bladder. Cystoscopy was used to remove the paper clips and to advance the battery into the bladder, allowing the removal of all objects, including the toothpaste caps, via cystoscopy. The patient was prescribed a one-week course of ceftriaxone for a UTI, and his hospital stay was complicated by the need for physical restraint to prevent further self-harm.

## Introduction

Lower urinary tract foreign body (FB) insertion is an uncommon presentation associated with diverse motivations, such as intoxication, autoeroticism, psychiatric illness, and perceived contraception [1]. Commonly inserted items include pencils, wires, batteries, animal parts, fluids, and other objects [2]. Some patients may be asymptomatic or experience minimal discomfort for extended periods. However, when symptoms do arise, they typically include hematuria, UTI, and severe pain [3]. An accurate history, physical examination, and imaging are essential for diagnosis. Conventional radiography is commonly employed, but early ultrasound can provide valuable guidance for management [4]. The approach to urethral FB removal depends on the object’s location and type and may involve endoscopic procedures, open surgeries, or catheterization. Here, we discuss a case involving a 51-year-old male who inserted paper clips, toothpaste caps, and a battery into his urethra.

## Case presentation

A 51-year-old male presented to the ED with dysuria after inserting an AAA battery and paper clips into his urethra. His past medical history was notable for post-traumatic stress disorder, antisocial personality disorder, and a previous history of self-mutilation requiring cystoscopy for removal. Upon arrival at the ED, urinalysis revealed pyuria, elevated red blood cells, proteins, and nitrites. He was started on a week of ceftriaxone for coverage of his UTI.

**Figure 1:**
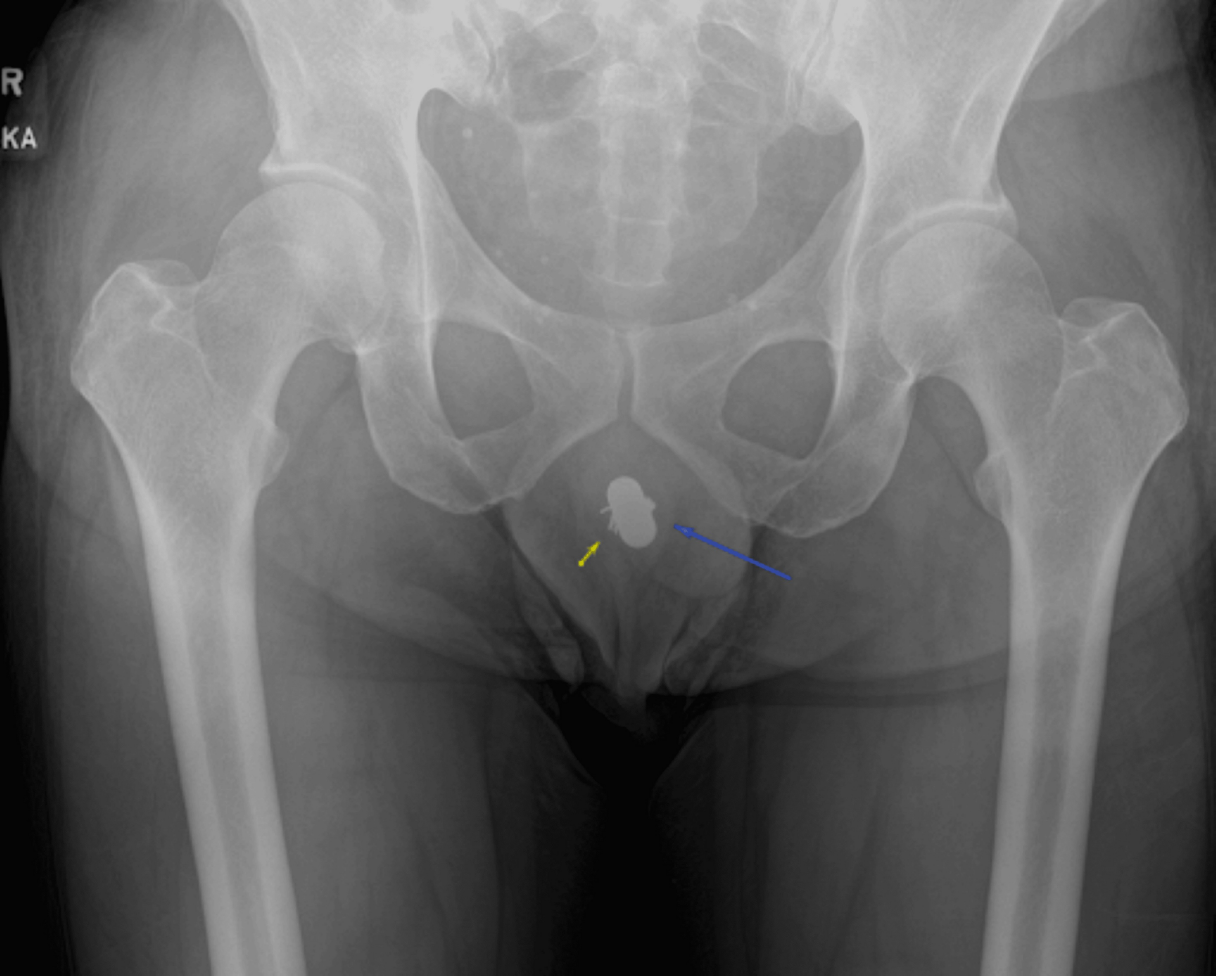
Pelvic X-ray demonstrating overlapping FBs, including a battery and multiple paper clips, located near the penile region FB, foreign body

**Figure 2:**
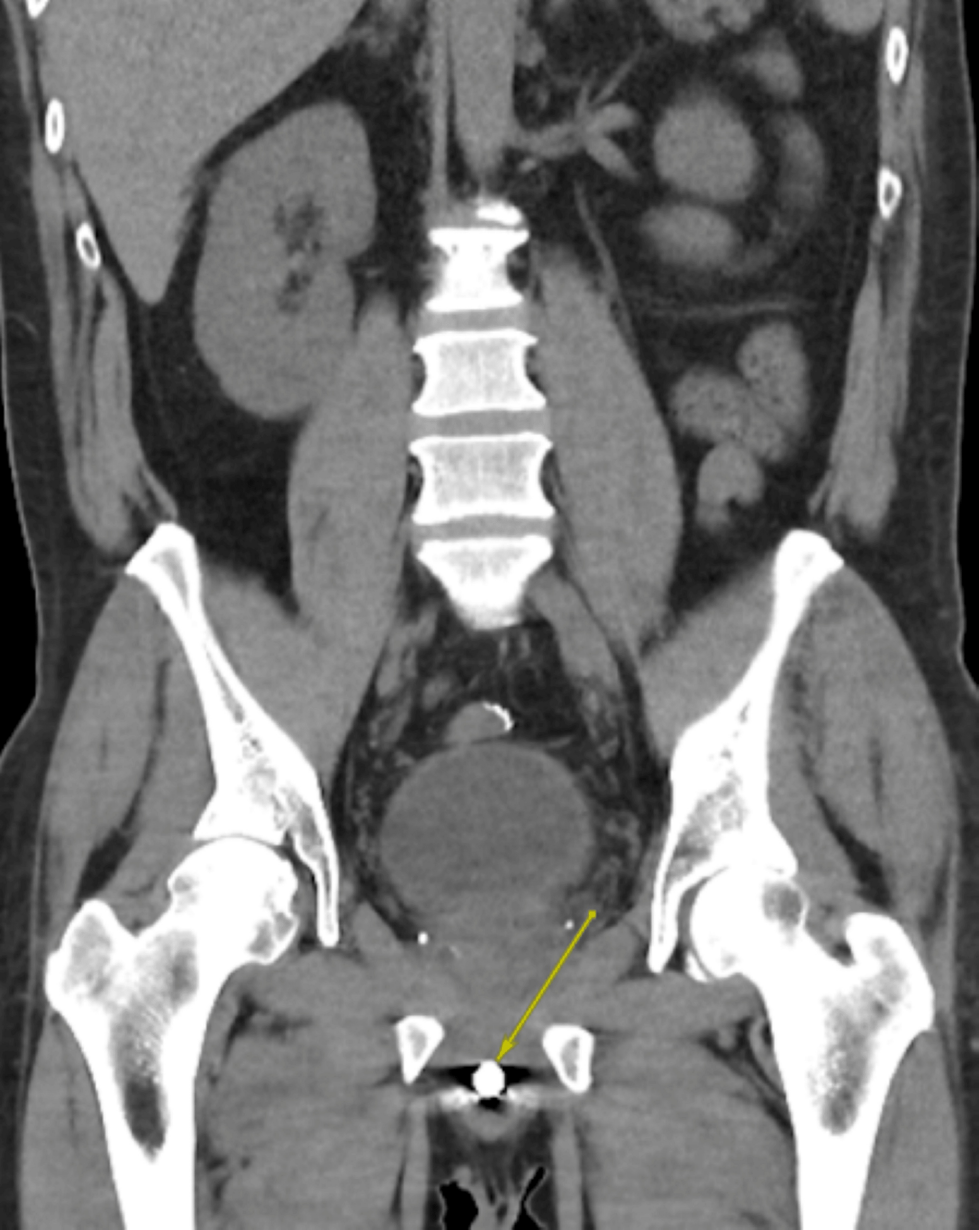
Coronal CT scan showing a hyperdense FB within the urethra, consistent with the cylindrical shape of a battery FB, foreign body

**Figure 3:**
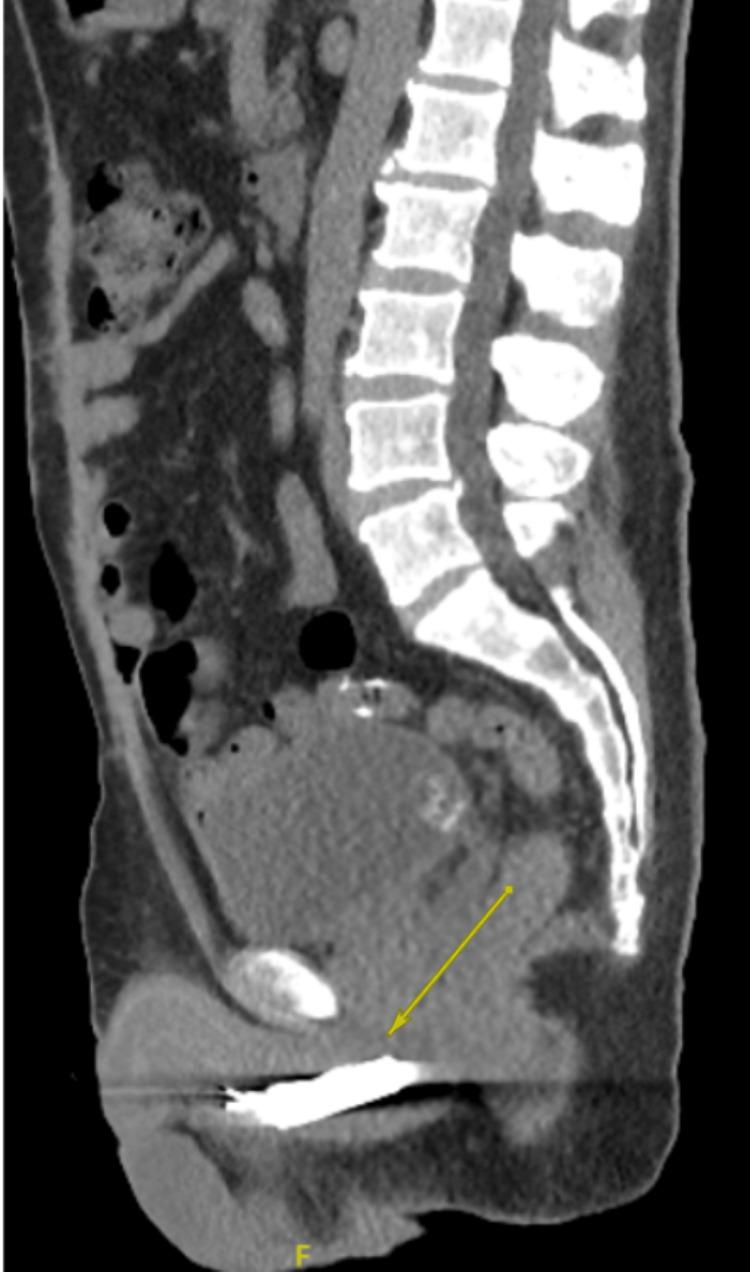
Sagittal CT scan showing a hyperdense, cylindrical object within the urethra, indicating self-insertion of a battery

**Figure 4:**
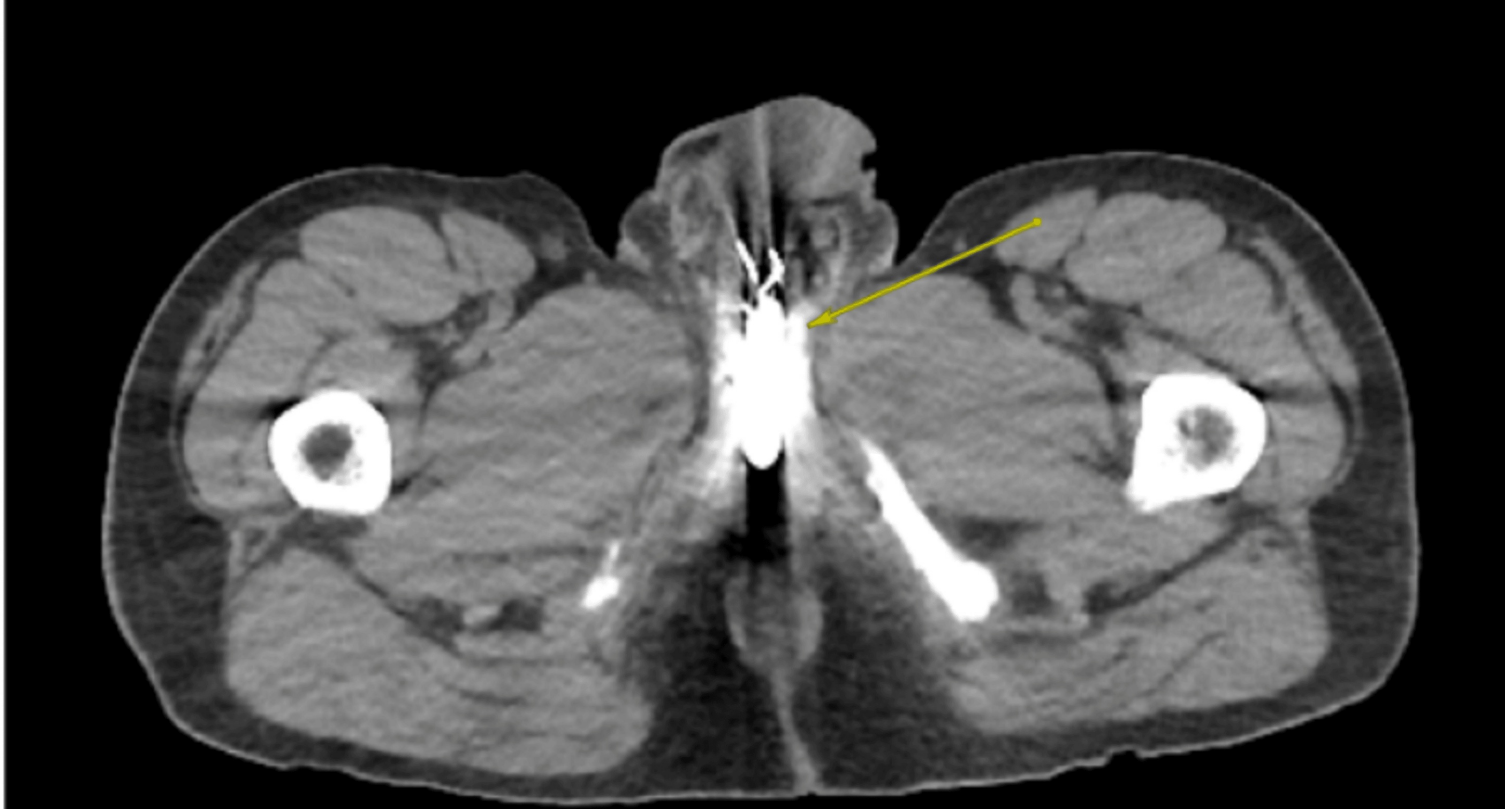
Axial CT scan showing hyperdense objects consistent with a battery and paper clips located within the urethra

**Figure 5:**
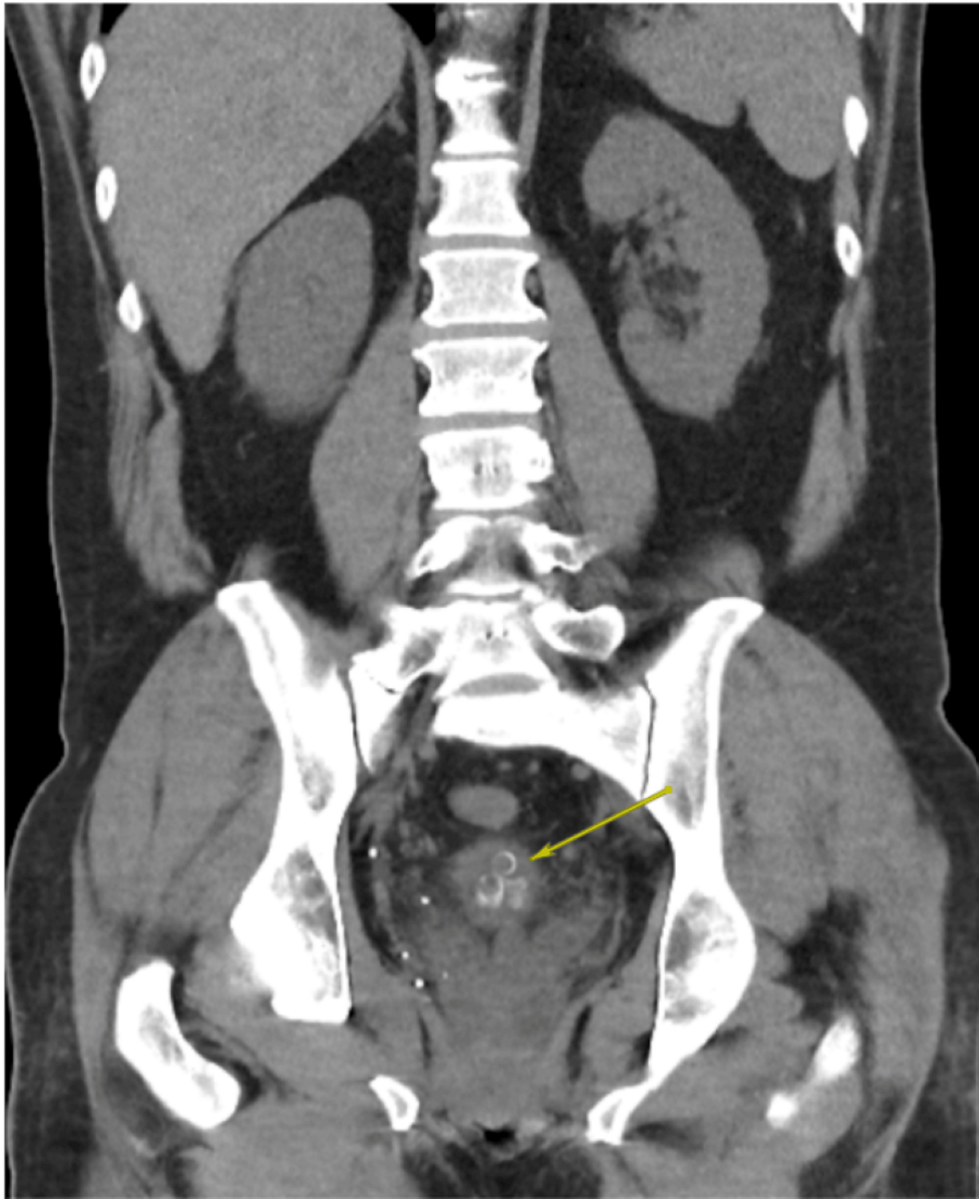
Abdominopelvic CT scan revealing multiple foreign objects within the bladder, with a hyperdense outline and central lucency consistent with toothpaste caps

**Figure 6:**
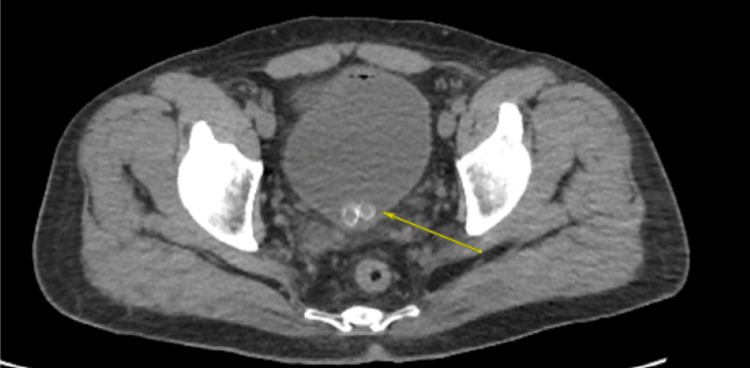
Axial CT scan showing two FB with central lucency, consistent with toothpaste caps located within the bladder FB, foreign body

A pelvic X-ray obtained in the ED showed evidence of two FBs overlapping the region of the penis (Figure 1). A CT scan of the abdomen and pelvis revealed no evidence of urethral perforation and showed a solid cylindrical radiopaque FB in the bulbar urethra, with a wire-like FB located just distally (Figure 2, Figure 3, Figure 4). Additionally, there were radiopaque structures in the bladder with central lucency consistent with toothpaste caps (Figure 5, Figure 6). No evidence of bladder perforation was found.

During the hospitalization, the patient repeatedly requested the use of four-point restraints to prevent his self-mutilation behaviors. Urology was consulted, and cystoscopy was performed for the removal of foreign objects. During the procedure, the paper clips were removed; however, significant damage to the urethra was noted due to the FBs. Attempts to remove the battery were unsuccessful, leading to its advancement into the bladder. Consequently, the decision was made to pursue cystoscopy to further remove the foreign objects. A Foley catheter was placed using a guidewire.

Cystoscopy was performed, during which the battery and toothpaste caps were successfully removed. A cystogram was conducted and showed no evidence of a leak. The Foley catheter was removed prior to discharge. A psychiatric consultation was obtained, and the patient’s discharge was delayed due to difficulties in finding appropriate placement, stemming from his behavior and the need for four-point restraints. The restraints were gradually tapered, along with the as-needed antipsychotic medication. Ultimately, he was discharged to a long-term psychiatric facility with a prescription for naltrexone to address impulsivity.

## Discussion

Lower urinary tract FB insertion is rare. However, one series review found that men are 1.7 times more likely to insert FBs into the urethra than women, with a mean age of presentation at 35.7 years old [2]. Another retrospective review of 35 cases involving 27 patients revealed a median age of 26 years, with 96% of the patients being men (26 men) compared to one woman (4%) [1]. While several motivations for self-insertion have been identified, one study found that 33% of cases were due to autoeroticism, 11% were psychiatric in nature, 39% were self-therapeutic, and 17% had no specific reason [5]. When symptoms do occur, patients may experience urethral, penile, or pelvic pain, bleeding at the urethral meatus, dysuria, and signs of sepsis [1].

When an FB is suspected in the lower urinary tract, determining its location and size is crucial for initial management. If the object is small, distally located, and palpable, radiographic imaging may not be necessary [1]. However, imaging is recommended to ascertain the size, location, and shape of the FB. Pelvic X-ray films are typically effective in detecting radiopaque objects, while ultrasound and CT are better for identifying non-opaque FBs [6]. Pelvic plain films usually suffice for initial detection, often followed by ultrasound or CT for further evaluation [7]. Plain films can detect approximately 80% of all FBs and up to 98% of radiopaque objects [8]. In a meta-analysis, ultrasound demonstrated a sensitivity of 72% and a specificity of 92% for FB identification [8]. If X-rays and ultrasound fail to identify a suspected FB, CT can be employed, as it is five to 15 times more sensitive than X-rays and excels in detecting plastic, glass, and stone objects [8].

The treatment of FBs in the urinary tract often depends on their size and location. For FBs measuring less than 1 cm that are palpable, mobile, and distally located without hematuria, manual sweeping of the urethra may be attempted as an initial removal strategy [1]. If manual removal is not feasible, endoscopic removal using instruments such as graspers and forceps is a common approach, as it minimizes urethral and bladder injuries and has a high success rate [7]. In cases where the FB is sharp or irregularly shaped, open surgery may be necessary [9]. Other surgical techniques include external urethrotomy and suprapubic cystostomy. FBs lodged in the penile urethra may require urethrotomy, while those within the bladder may necessitate suprapubic cystostomy [10]. An alternative technique involves using a 6-French pediatric Foley catheter; inflating the balloon with 0.5-2 cc of fluid can help minimally dilate the urethra to facilitate the passage of the object, thereby reducing urethral trauma from forceps and graspers [4]. Many of these techniques require significant skill, necessitating urology consultation.

Prompt diagnosis and intervention are essential to prevent complications associated with FB insertion. Persistent FBs can lead to infections, fistula formation, diverticula, or stones, with some cases resulting in serious, life-threatening complications such as uremia and sepsis [7,11]. Other potential complications include erectile dysfunction, strictures, and incontinence [4]. The severity of complications depends on the depth of the FB, the removal strategy employed, and the duration it remains in the body. The urethra has a thin, highly vascularized submucosal layer, making antibiotic prophylaxis necessary [4,9]. Additionally, tetanus prophylaxis is warranted after FB removal [9]. Timely management is crucial to mitigate and avoid further complications from an FB within the urinary tract.

## Conclusions

Although uncommon, lower urinary tract FB insertion occurs when patients insert items such as batteries, pencils, wire-like objects, and other FBs into their urethra. This behavior may stem from psychiatric illness, autoeroticism, intoxication, or perceived contraception. Symptoms can vary widely, but common presentations include pain, UTIs, and hematuria. A thorough physical examination and appropriate imaging are crucial for an accurate diagnosis. Pelvic X-ray is typically the initial imaging study, followed by CT, which is more effective at detecting non-opaque foreign objects. Treatment approaches depend on the size and location of the FB and may include manual urethral sweeping, endoscopic removal, or open surgery. Timely diagnosis and intervention are essential to prevent potentially life-threatening complications, including sepsis and uremia. Additionally, inpatient psychiatric consultation may be beneficial, particularly for patients exhibiting self-mutilation behaviors, as it facilitates management strategies in both inpatient and outpatient settings aimed at reducing these harmful actions.
